# *Streptococcus pneumoniae* and Its Virulence Factors H_2_O_2_ and Pneumolysin Are Potent Mediators of the Acute Chest Syndrome in Sickle Cell Disease

**DOI:** 10.3390/toxins13020157

**Published:** 2021-02-17

**Authors:** Joyce Gonzales, Trinad Chakraborty, Maritza Romero, Mobarak Abu Mraheil, Abdullah Kutlar, Betty Pace, Rudolf Lucas

**Affiliations:** 1 Department of Medicine, Medical College of Georgia at Augusta University, Augusta, GA 30912, USA; akutlar@augusta.edu (A.K.); rlucas@augusta.edu (R.L.); 2 Institute for Medical Microbiology, Justus-Liebig-University, Biomedical Research Centre Seltersberg, 35392 Giessen, Germany; trinad.chakraborty@mikrobio.med.uni-giessen.de (T.C.); Mobarak.Mraheil@mikrobio.med.uni-giessen.de (M.A.M.); 3 Vascular Biology Center, Medical College of Georgia at Augusta University, Augusta, GA 30912, USA; mromerolucas@augusta.edu; 4 Department of Pharmacology and Toxicology, Medical College of Georgia at Augusta University, Augusta, GA 30912, USA; 5 Department of Pediatrics, Medical College of Georgia at Augusta University, Augusta, GA 30912, USA; bpace@augusta.edu

**Keywords:** sickle cell disease, acute chest syndrome, *Streptococcus pneumoniae*, pneumolysin, H_2_O_2_

## Abstract

Sickle cell disease (SCD) is one of the most common autosomal recessive disorders in the world. Due to functional asplenia, a dysfunctional antibody response, antibiotic drug resistance and poor response to immunization, SCD patients have impaired immunity. A leading cause of hospitalization and death in SCD patients is the acute chest syndrome (ACS). This complication is especially manifested upon infection of SCD patients with *Streptococcus pneumoniae* (*Spn*)—a facultative anaerobic Gram-positive bacterium that causes lower respiratory tract infections. *Spn* has developed increased rates of antibiotics resistance and is particularly virulent in SCD patients. The primary defense against *Spn* is the generation of reactive oxygen species (ROS) during the oxidative burst of neutrophils and macrophages. Paradoxically, *Spn* itself produces high levels of the ROS hydrogen peroxide (H_2_O_2_) as a virulence strategy. Apart from H_2_O_2_, *Spn* also secretes another virulence factor, i.e., the pore-forming exotoxin pneumolysin (PLY), a potent mediator of lung injury in patients with pneumonia in general and particularly in those with SCD. PLY is released early on in infection either by autolysis or bacterial lysis following the treatment with antibiotics and has a broad range of biological activities. This review will discuss recent findings on the role of pneumococci in ACS pathogenesis and on strategies to counteract the devastating effects of its virulence factors on the lungs in SCD patients.

## 1. Introduction

Sickle cell disease (SCD) arises from homozygous single nucleotide polymorphisms (SNP) in the sixth codon of the β-globin gene (HbSS) on chromosome 11 which causes substitution of a single valine (Val) residue for glutamic acid (Glu) [1]. As a consequence, HbSS can polymerize under deoxygenation and mediates the sickling of erythrocytes [2].

SCD represents one of the most common autosomal recessive disorders in the world. In the US, 8% of African Americans are heterozygous and have the sickle cell trait, whereas 1 in 600 is homozygous and has sickle cell disease [3]. A leading cause of death and hospitalization in patients with SCD is the acute chest syndrome (ACS) [4] characterized by abnormal interactions between sickle erythrocytes and/or platelets and the vascular endothelium [5].

Although both infectious and non-infectious causes for ACS have been suggested, the pathophysiology of the disease remains elusive and is often ill-defined, even at autopsy. [6] There are multiple identified etiologies associated with the development of ACS, but bacterial and viral infection represents the main cause. Standard therapy of ACS remains supportive and includes transfusion (exchange or simple), use of bronchodilators and mechanical ventilation.

### 1.1. Streptococcus pneumoniae: A Prominent Etiological Agent of Severe Pneumonia

Infections of the lower respiratory tract represent the most common cause of infectious disease mortality and the fifth highest cause of death overall. With 294,000 deaths in infants under 5 years old in 2015, *Spn* represents the leading cause of death in infants worldwide [7] and is the major etiologic agent of community-acquired pneumonia. Elderly and immunocompromised patients are also more susceptible to *Spn* [8]. *Spn* is a facultative anaerobe Gram-positive bacterium that colonizes the upper respiratory tract as a commensal bacterium in healthy individuals. This asymptomatic carriage phase is considered a prerequisite for subsequent development of pneumonia, which can occur once the pathogen migrates into the lungs. A retrospective study showed that *Spn* was the most common bacterial co-infection in COVID-19 patients [9].

### 1.2. Increased Susceptibility to Pneumococcal Infections in SCD Patients

Patients with SCD, especially children, are particularly prone to invasive infections [10] and this can promote ACS. SCD patients with pneumococcal pneumonia are sicker than non-SCD patients with a similar infection, as demonstrated by longer duration of fever and hospitalization, an increased need for red blood cell transfusion and the presence of pleural effusions [11]. Children and infants with SCD are extremely susceptible to bacteremia, pneumonia and meningitis caused by *Spn* and have a 100-times higher rate of *Spn* infection than non-SCD infants and children. This vulnerability is at least partially due to functional asplenia in infants with SCD [12]. Other contributions of increased susceptibility to pneumococcal disease in children and adults with SCD are a dysfunctional IgG and IgM antibody response, a lack of splenic clearance and defects in alternative pathway fixation of complement and opsonophagocytic dysfunction. All of these contribute to the susceptibility of infection from all polysaccharide-encapsulated bacteria including pneumococci, *Neisseria meningitides* and *Hemophilus influenzae* [10,13]. *Spn* was responsible for about half of the episodes of pneumonia in SCD patients prior to prophylactic penicillin in children and application of the pneumococcal vaccines at all ages [14].

At initial diagnosis, a case of pneumococcal pneumonia is often indistinguishable in the sickle cell patient and the patient that has normal immunity. However, the patient with SCD may soon develop ACS, defined as a new pulmonary infiltrate, often accompanied by fever, chest pain, cough, wheezing and tachypnea. A published case study, which points out the vulnerability of a patient with SCD and functional asplenia with decreased immunity, reports on a 22-year old African American male with hemoglobin SD disease (a type of SCD) admitted to the hospital with Gram-positive meningitis, sepsis, pneumonia, and vaso-occlusive pain crisis. He was initially diagnosed and treated for meningitis with powerful antibiotics, ceftriaxone, and vancomycin. Four days after admission his respiratory status progressively worsened and he was diagnosed with pneumonia due to *Spn*. His vaccine status was up-to-date, and it was suspected that he could have an immune deficiency or inadequate specific IgG levels against some strains of the pneumococcal polysaccharide vaccine 23 (PPSV23). Two months after his hospitalization he received his fourth PPSV23 booster, which produced normal protective levels of IgG. This case highlights the uncertainty about the effectiveness of pneumococcal vaccination and more specifically in patients with functional asplenia, as can occur in SCD [15].

An early study by Overturf et al. reported mortality in a vaccinated (Pneumovax 14-valent vaccine) 37-month-old boy with SS (homozygous disease), who had sepsis and died within hours after the onset of symptoms. *Spn*, Type 6, was isolated from antemortem blood cultures. In this case, serum immunoglobulins were normal but titers to Type 6 were low [16]. Ahonkhai and colleagues published a two-case series of pediatric patients who contracted *Spn* and invasive pneumococcal disease (IPD) after vaccination with the 14-valent pneumococcal vaccine [17]. Later studies postulated that abnormalities of immunologic defense mechanisms, including synthesis of polyclonal IgG and IgM, the alternative complement pathway, opsonic activity and T and B cell interaction exist in the patient with SS genotype [18,19]. Another 10-case report evaluated IPD in vaccinated patients [20]. Among the cases was a 16-year-old male with SS disease, who had received two doses of PPSV23 and one dose of PCV7 (the precursor to PCV-13). He was admitted to the intensive care unit with hypotension and acute respiratory failure. In his case, as in the other nine cases, the severe illness was from serotypes of *Spn* not contained in the vaccines he received [20]. A 2016 study, evaluating the vaccine response in a single Sickle Cell Center, found that only 36% of patients had protective levels of anti-pneumococcal antibody titers at 37 months after vaccination. Among the group of patients who had sub-therapeutic titers, 64% demonstrated a vaccine response to less than 25% of the tested serotypes. The study team concluded that anti-pneumococcal immunity may not be optimal in the current vaccine strategy, leaving SCD patients vulnerable to IPD [21].

Vaccinated patients might carry a lower risk of infection, however, vaccination by itself should never allow a false sense of security [22]. The rapidity of an overwhelming pneumococcal strep infection is well known. When not recognized early or when the patient does not receive early and aggressive medical care, mortality rates can range from 50 to 70%. This mortality may however decrease to 10% if recognized early and treated aggressively [23]. In conclusion, irrespective of the vaccination status, SCD patients who all have functional asplenia and a potential pneumococcal infection of any type, including ACS, should receive immediate and aggressive care [23].

## 2. Prevention and Treatment of Pneumococcal Pneumonia

Health maintenance of children with SCD includes the pneumococcal vaccines and prophylactic antibiotics such as amoxicillin for pneumococcal infection until the child is five years old and azithromycin or clarithromycin for ages over five [24]. Recent data from murine models, however, suggest that antibiotic therapy needs to be initiated prior to breakdown of the alveolar-capillary barrier and systemic inflammation in pneumococcal pneumonia [25].

Currently, two main types of vaccines have been developed to reduce or eliminate the burden of pneumococcal infections: the unconjugated 23-valent polysaccharide vaccine and the 10- or 13-valent conjugated polysaccharide vaccine. Their coverage of all serotypes is however not universal, and they fail to affect non-capsulated pneumococci. Treatment of pneumococcal pneumonia in susceptible populations, such as SCD patients, is problematic as strains of pneumococci have become increasingly resistant and difficult to treat with the traditional antibiotic treatment, such as penicillin. Multidrug-resistant strains of pneumococci have become increasingly established due to the widespread availability and overuse of antibiotics; consequently pneumococcal infections remain a worldwide public health problem [26]. There is growing concern that a major problem in the treatment of *Spn* in ACS is the further evolution of antibiotic resistant strains. The high death rate associated with pneumococcal disease and the emergence of antibiotic resistance and the problems associated with current vaccines have stimulated interest in the mechanisms of virulence.

Universal immunization of infants and toddlers against pneumococcus has dramatically altered the landscape of pneumococcal disease and there is a decrease in invasive pneumococcal disease [27]. However children with any comorbid conditions have higher rates of pneumococcal disease and increased case fatality rates compared with otherwise healthy children [27]. Protection will continue to require strategies to address the increased susceptibility in these children.

## 3. H_2_O_2_: A Pneumococcal Virulence Factor That Can Aggravate ACS

Vascular damage in ACS is at least partially mediated by generation of oxygen-related molecules, such as superoxide (O_2_^−^), hydrogen peroxide (H_2_O_2_), peroxynitrite (ONOO^−^), and the hydroxyl (•OH) radical [5]. Enhanced intravascular hemolysis can lead to an accumulation of hemoglobin (Hb) and heme in plasma, which in turn decreases bioavailability of nitric oxide (NO) and enhances ROS generation [2]. Moreover, the continuous oxidation of the heme iron in Hb (auto-oxidation) and impaired anti-oxidant capacity promote a pro-oxidative environment in sickle erythrocytes, which compromises overall redox balance in these cells and affects their metabolic state. Ferrous HbS (HbS (Fe^2+^)) auto-oxidizes to the non-functional ferric (Fe^3+^) (metHb) form at a nearly double rate as normal Hb (HbA), despite the presence of reducing enzymes. This causes a considerable metHb accumulation inside the erythrocytes. Hence, SCD patients have a much higher propensity for ROS generation than individuals without SCD [28,29].

During periods of reperfusion following vaso-occlusive crisis (VOC), increased generation of ROS can mediate activation of second messengers. A prominent one among these is nuclear factor-κB (NF-κB), which mediates the upregulation of endothelial adhesion molecules, such as vascular cell adhesion molecule 1 (VCAM-1), intercellular adhesion molecule 1 (ICAM-1) and especially P- selectin [6,30]. These adhesion molecules in turn facilitate binding of sickle RBCs and leukocytes to vascular endothelium, which promotes the development of VOC [5]. However, ROS can also directly injure the endothelium by peroxidation of the lipid membrane, inducing DNA fragmentation or disrupting adherens and tight junctions [31]. Knockout of the transcription factor NRF2, a master regulator of the cellular oxidative stress response and activator of fetal hemoglobin expression, was shown to reduce expression of its target anti-oxidant proteins and increase generation of ROS, pro-inflammatory cytokines as well as to promote expression of adhesion molecules in SCD mice [32,33].

In pneumococcal infection, and thus also likely in pneumococcal pneumonia-associated ACS, H_2_O_2_ plays a highly complex role in the development of acute lung injury. On the one hand, this ROS is involved in anti-bacterial defense mechanisms mediated by neutrophils and alveolar macrophages, but on the other hand it can significantly contribute to alveolar-endothelial barrier dysfunction and to the impairment of alveolar liquid clearance mechanisms. Alveolar liquid clearance is mainly mediated by vectorial Na^+^ transport in type 1 and 2 alveolar epithelial cells [34].

Of note, pneumococci can endogenously generate up to millimolar levels of H_2_O_2_ as a by-product during aerobic growth, with the enzymes pyruvate oxidase and lactate dehydrogenase as the main sources for this production [35]. Endogenously generated H_2_O_2_ can rapidly diffuse through cell membranes and will accumulate in the extracellular milieu of pneumococci in concentrations high enough to kill or inhibit other pathogens [36]. Hence, high levels of endogenously generated H_2_O_2_ provide a competitive advantage over other pathogens and commensal bacteria in the respiratory tract.

In order to allow for efficient gas exchange, a tight barrier structure of only 0.3 μm thick separates the capillary blood from alveolar gas [31,37]. A disruption of the alveolar- capillary barrier, as is found in ACS, can result in alveolar flooding, which in turn impairs alveolar gas exchange. As a consequence, hypoxia and hypercapnia can arise, both of which are hallmarks of the acute respiratory distress syndrome (ARDS). During pneumococcal pneumonia, high H_2_O_2_ levels can accumulate in the alveolar space, mainly originating from pneumococcal secretion, but to a lesser extent also from inflammatory neutrophils and alveolar macrophages, which first generate superoxide from NADPH oxidase 2 (Nox-2). Superoxide is then further converted into H_2_O_2_ by superoxide dismutase. Intravascular H_2_O_2_ challenge in intact lungs was shown to cause both capillary endothelial and alveolar epithelial barrier dysfunction [38].

Apart from inducing oxidative stress in resident pulmonary cells, *Spn* was also shown to induce endoplasmic reticulum (ER) stress in club cell-like H441 lung epithelial cells following infection. Pneumococcal H_2_O_2_, was demonstrated to be the main mediator of the pneumococcal induction of ER stress [39].

In conclusion, the pneumococcal virulence factor H_2_O_2_ can be generated in the micro-molar range by the Nox-2/myeloperoxidase or Nox2/superoxide dismutase pathways in neutrophils and alveolar macrophages, respectively and in millimolar quantities by the pyruvate oxidase pathway in pneumococci. Endogenously generated H_2_O_2_ exerts mainly positive actions in pneumococci, including the killing of competing respiratory tract pathogens, increasing virulence, and promoting biosynthetic pathways. By contrast, although neutrophils and macrophage-derived H_2_O_2_ promotes bacterial clearance, excessively high concentrations of H_2_O_2_ in the host’s alveolar space, mainly of pneumococcal origin, can promote apoptosis, induce the unfolded protein response ER stress [40], increase inflammation and impair barrier function in alveolar epithelial and capillary endothelial cells.

As SCD patients have a much higher propensity for ROS generation than individuals without SCD, due to continuous auto-oxidation of the heme iron in hemoglobin S (HbS) and impaired anti-oxidant capacity in erythrocytes, they will be particularly susceptible to increased H_2_O_2_ concentrations of pneumococcal origin and can experience a dramatic disruption of the alveolar-capillary barriers.

## 4. Pneumolysin: A Pneumococcal Virulence Factor with Potent Cytotoxic Activity in ACS

Two pneumococcal exotoxins—pneumolysin (PLY) and autolysin—have been implicated in the development of lung injury in pneumococcal bacterial infections [40]. In this review we focus principally on pneumolysin (PLY). Pneumolysin (PLY) is a virulence factor produced by *Spn* with a molecular weight of 53 kDa, composed of 471 amino acids. PLY is a cytolysin common to all serotypes of *Spn* [41] which lacks a typical secretory signal. Therefore, in contrast to other characterized cytolysins, PLY can be localized in the cytoplasm or in the pneumococcal cell wall [42] and released during bacterial growth and autolysis [43]. As such, PLY can reach and destroy host tissues when pneumococci break down and when the bacterium comes in close contact with the host cell. PLY is essentially a cholesterol-binding exotoxin with a broad range of biological activities, including the ability to lyse and interfere with the function of cells and soluble molecules of the immune system [44]. PLY directly impairs alveolar epithelial and endothelial barrier function by affecting myosin light chain (MLC) phosphorylation and on microtubule acetylation [45,46,47,48] directly through its pore-forming activity, rather than indirectly through stimulation of leukocyte infiltration and cytokines.

PLY is a member of the cholesterol-dependent cytolysin family, a large group of proteins that attack cholesterol-containing membranes, forming ring shaped pores that mediate cell death [49,50]. Binding of PLY to cholesterol is followed by oligomerization [51,52]. When a pore is formed in the cell membrane, the tight regulation of what can and cannot enter/leave a cell is disrupted and the in- and efflux of ions, such as Ca^2+^ and small molecules, such as amino acids and nucleotides within the cell is perturbed and water from the surrounding tissue can enter. This loss of important small molecules to the cell disrupts protein synthesis and impairs crucial cellular reactions. The continuous entry of water into the cell can cause uncontrolled cell swelling. This results in the distortion of large parts of the cell membrane which gives way under the mounting internal pressure. In the end, the cell can erupt [53].

PLY also interferes with the function of cells in the immune system [34]. In mouse models of *Spn*, PLY has been found in experimentally induced mouse lungs infected with *Spn* and it has been suggested to stimulate lethal inflammation in murine lung tissues [36].

PLY can induce apoptosis in respiratory epithelium, endothelium, and neuronal cells [54]. Although antibiotics to treat pneumococcal infection, like ceftriaxone, are widely available, mortality and morbidity can still occur, and are related to the sudden release of high levels of pneumolysin in the lung compartment and its subsequent cellular pathogenicity. Indeed, PLY is not actively secreted from the intact cell, but is rather released by degradation of the bacterial cell wall during cell-division, autolysis or following antibiotic action [54]. One study showed that during the first 3 h after addition of antibiotics, concentrations of PLY in cultures incubated with ceftriaxone tended to be higher than in control cultures [55]. PLY disrupts the host immune defenses against infection by multiple effects, including the known cellular pore formation, inflammation, barrier dysfunction, complement activation, apoptosis of non-immune cells and by targeting host factors affecting infection.

PLY is associated with the phosphorylation and activation of the p38 mitogen-activated protein kinase (MAPK) pathway. This was proposed to negatively affect apoptosis in endothelial cells and neurons [56,57]. Pneumolysin also selectively regulates expression of MAPK phosphatase 1 (MKP1), which inhibits the p38 signaling pathway [58]. This causes upregulation of β-defensin 2, which mounts an effective defense against *S. pneumoniae* infection [58].

However, p38 has also been proposed to play a protective role in PLY action. Plasminogen activator inhibitor (PAI-1) is required for recovery from local tissue injury and is induced by MAPK kinase 3 (MMK3) and p38. MKK3/p38 induced PAI-1 is required in vivo for normal host survival upon infection with pneumococci expressing PLY and upon treatment with purified PLY, which indirectly supports an in vivo role for p38 in PLY defense [59].

Another protective facet of p38, when activated, is induced by some medications used in SCD patients. Numerous pharmacologic agents induce fetal hemoglobin (HbF) synthesis [60], these include histone deacetylase (HDAC) inhibitors, hydroxyurea and valproic acid which all converge at the p38 MAPK pathway to induce HbF expression. Valproate acid used in the treatment of seizure disorders increased HbF levels in 33% of patients [61]. P38 MAPK expression in human chronic myelogenous leukemia K562 cell lines increases γ-globin synthesis. A study using K562 erythrocyte cells showed that the induction of HbF synthesis by valproate increased activation of the p38 pathway contributing to the HbF inducing activity of valproate [60,62]. Taken together, the p38 pathway can have an important role in protection from pneumococcal and PLY-induced pathology and in induction of protective HbF in SCD.

Another enzyme induced by PLY is the deubiquitinating enzyme cylindromatosis (CYLD), a regulatory enzyme required for T cell development, tumor cell proliferation and nuclear factor kappa B (NF-κB) transcription factor signaling. CYLD plays a potent role in bacteria-induced lethality. CYLD appears to be involved in PLY-induced microvascular barrier dysfunction. Indeed, CYLD deficiency protected mice from PLY-induced acute lung injury and mortality. CYLD was highly induced by PLY and it inhibited MKK3-p38 induced expression of PAI-1 in the lung potentiating ALI [63,64].

An additional property of PLY that contributes to the virulence of PLY is its ability to activate the complement pathway [49]. The complement “defense system” consists of a number of small proteins found in the blood that are predominantly synthesized by the liver and circulate inactively in the blood until needed for response to an insult in the body such as a bacterial infection. It is known that patients with complement system defects, liver cirrhosis, or functional asplenia, such as patients with SCD, are more susceptible to pneumococcal infections. PLY released during pneumococcal autolysis activates complement distally from the organisms, an activity thought to contribute to inflammation and virulence [65]. It is postulated that PLY by activating classical complement consumes complement at a distance from the surface of intact organisms, reducing available opsonins necessary for their uptake and killing by phagocytes [59]. The induction of the complement system can also damage the host due to an overwhelming inflammatory response [66]. Additionally, the effect of PLY on the circulating complement proteins diminishes their levels, leaving the host with a weakened defense. In a study comparing susceptibility of cirrhotic *versus* control rats to pneumococcal infection, levels of complement were found to be significantly reduced in the cirrhotic group. This significantly prevented effective opsonizing activity and phagocytosis in the cirrhotic rats. This study suggests that PLY has a significant negative effect on serum complement levels and complement activity [65].

Invasive pneumococcal disease develops when inflammation promotes receptor-mediated translocation of bacteria from the alveoli into the bloodstream. This invasive process involves two steps. First, pneumococci attach to host cells using specific adhesins which are virulence factors. Following adherence, bacteria bind to phosphorylcholine that decorate the receptor for platelet-activating factor (PAF), a circulating lipid chemokine that crosses the host cell by receptor-mediated endocytosis [51,67,68]. Pneumococci utilize PAF-R interaction for invasion into host cells, and mice lacking PAF-R show a dramatic attenuation of invasive pneumococcal disease [68]. PAF receptor expression increases with aging and this contributes to increased vulnerability of the elderly to pneumococcal pneumonia [69]. PAFRs are also found to be upregulated in mouse models of SCD and although defective splenic clearance and asplenia is known to cause vulnerability of SCD patients to *Spn*, this represents another major contributing factor [66,67,70]. A key role for the PAF-R in *Spn* infection has also been suggested in perfused mouse lungs. PLY caused a dose- dependent increase in pulmonary vascular resistance and in pulmonary microvascular leakage, which was significantly diminished in lungs of PAF-R-deficient mice [71].

Statins (3-hydroxy-3-methylglutaryl coenzyme A reductase inhibitors) lower circulating lipid levels and are very effective in decreasing the risk of cardiovascular disease. In a mouse SCD model, statins were shown to reduce PAF-R expression in alveolar epithelium and vascular endothelium. The decrease in PAF-R expression was associated with reduced bacterial adherence and invasion. Statins were moreover shown to prevent cytolysis of endothelial cells (but not erythrocytes) treated with purified PLY. One mechanism that was proposed is that statins interfere with lipid raft formation, which are required for PLY-dependent pore formation and subsequent cytotoxic effects [72]. However, in contrast to these findings in SCD mouse models, statins were shown to be detrimental for pneumococcal infection in obese mice [72], which raises questions on their overall therapeutic potential in treating pneumococcal pneumonia.

Apart from PLY, another major pneumococcal virulence factor is N-acetylmuramoyl-L-alanine amidase, a toxin known as autolysin (LytA). LytA is found in the cytoplasm of pneumococci. Its pathologic action occurs when it then binds and degrades the cell wall of neighboring cells, as such instigating a destructive lytic cascade [73]. PLY can also be released by the action of LytA. Activation of LytA may be triggered by the immune system, bacterial virulence factors or antibiotics [73]. It was at one time, believed that LytA autolysis was necessary to release PLY from the cytoplasm to the extracellular milieu. However, studies have shown that PLY and LytA may sometimes be independent virulence factors of pneumococci [72]. More recent research has shown that PLY and LytA are found in chromosomally-located pathogenicity islands [74].

Pathogenicity islands are a distinct class of genomic islands acquired by microorganisms through horizontal gene transfer. They encode virulence factors of pathogenic bacteria and play a crucial role in the evolution of a broad spectrum of bacteria including antibiotic resistance and virulence [75].

## 5. Conclusions

Pneumococcal infection can have serious implications in pneumonia and sepsis. Patients with SCD who have functional asplenia and increased oxidative stress in the vasculature, are at great risk for pneumococcal infections from infancy through to their adult lives. A major virulence factor of pneumococcal infection, PLY is a major contributor of pathogenicity. PLY is released early in infection and during antibiotic therapy contributing to the formidable morbidity and mortality of this disease. Pneumococcal vaccines are recommended in SCD patients, but their highly increased vulnerability to pneumococcal disease stresses the need for early diagnosis and intervention. Future studies should further address mechanisms that account for increased susceptibility of SCD patients to pneumococcal pneumonia-associated ACS and the role of pneumococcal virulence factors, mainly PLY and H_2_O_2_, in this process.
